# Comparative actions of progesterone, medroxyprogesterone acetate, drospirenone and nestorone on breast cancer cell migration and invasion

**DOI:** 10.1186/1471-2407-8-166

**Published:** 2008-06-09

**Authors:** Xiao-Dong Fu, Maria Silvia Giretti, Lorenzo Goglia, Marina Ines Flamini, Angel Matias Sanchez, Chiara Baldacci, Silvia Garibaldi, Regine Sitruk-Ware, Andrea Riccardo Genazzani, Tommaso Simoncini

**Affiliations:** 1 Molecular and Cellular Gynecological Endocrinology Laboratory (MCGEL), Department of Reproductive Medicine and Child Development, University of Pisa, Pisa 56100, Italy; 2 Population Council and Rockefeller University, New York, USA

## Abstract

**Background:**

Limited information is available on the effects of progestins on breast cancer progression and metastasis. Cell migration and invasion are central for these processes, and require dynamic cytoskeletal and cell membrane rearrangements for cell motility to be enacted.

**Methods:**

We investigated the effects of progesterone (P), medroxyprogesterone acetate (MPA), drospirenone (DRSP) and nestorone (NES) alone or with 17β-estradiol (E2) on T47-D breast cancer cell migration and invasion and we linked some of these actions to the regulation of the actin-regulatory protein, moesin and to cytoskeletal remodeling.

**Results:**

Breast cancer cell horizontal migration and invasion of three-dimensional matrices are enhanced by all the progestins, but differences are found in terms of potency, with MPA being the most effective and DRSP being the least. This is related to the differential ability of the progestins to activate the actin-binding protein moesin, leading to distinct effects on actin cytoskeleton remodeling and on the formation of cell membrane structures that mediate cell movement. E2 also induces actin remodeling through moesin activation. However, the addition of some progestins partially offsets the action of estradiol on cell migration and invasion of breast cancer cells.

**Conclusion:**

These results imply that P, MPA, DRSP and NES alone or in combination with E2 enhance the ability of breast cancer cells to move in the surrounding environment. However, these progestins show different potencies and to some extent use distinct intracellular intermediates to drive moesin activation and actin remodeling. These findings support the concept that each progestin acts differently on breast cancer cells, which may have relevant clinical implications.

## Background

Hormone replacement therapy (HRT) is used to relieve menopausal symptoms and to protect postmenopausal women from osteoporosis [1]. Progestins are required in HRT in women to prevent an inappropriate estrogen-dependent endometrial proliferation. A variety of progesterone receptor agonists, including natural progesterone (P) or synthetic progestins are commonly used in HRT regimens [2]. However, different clinical trials, particularly the Million Women Study and the Women's Health Initiative trial, have reported increases in breast cancer risk associated with progestin use in HRT [3-5], suggesting a deleterious role of progestins on breast cancer.

The pharmacological properties of progestins vary depending on the parent molecule from which they are derived, leading to considerable variations of the full spectrum of biological activities [2,6]. For instance, beyond the obvious progestogenic activity, medroxyprogesterone acetate (MPA), a derivative of 17-hydroxyprogesterone, is also endowed with glucocorticoid activity [2].

These pharmacological discrepancies may account for the diverse impact of progestins on breast cancer development and progression. For instance, the French cohort study as well as the E3N-EPIC cohort study show that continuous-combined HRT with synthetic progestins is associated with an increased relative risk of breast cancer in postmenopausal women, but this is not found with HRT containing natural progesterone [7,8]. Hence it would be clinically important to be able to differentiate the effects on breast cells of the different progestins used for HRT.

In the past few years progestins with improved receptor-selectivity profiles have been introduced into clinical practice. Drospirenone (DRSP), a progestogen derived from spirolactone, is characterized by significant anti-androgenic and anti-minineralocorticoid activities [9]. Due to this, DRSP administration in HRT helps to prevent sodium and water retention as well as body weight increases in normotensive post-menopausal women and to decrease blood pressure in patients with mild hypertension [10]. Another new progestin with specific characteristics is nestorone (NES), a 19-nor derivative of progesterone. NES is characterized by a strong progestational activity, combined with a complete lack of androgenic, estrogenic, and glucocorticoid-like activities. This makes the compound well-tolerated and devoid of side effects in clinical practice [11].

The main cause of morbidity and mortality in breast cancer patients is the spread to the lymph nodes and to distant organs of tumor cells [12,13]. While a lot is known on the effects and mechanisms of action of progesterone on breast cancer cell proliferation [14-16], limited information is available on the impact on cell migration and invasion. Moreover, the actions of the new progestins (such as DRSP or NES) on breast cancer have not been investigated.

Cell migration and invasion are based on a complex and dynamic set of morphological cellular changes, primarily including the reorganization of the actin cytoskeleton [17]. During cell movement, the cytoskeletal actin fibres are dynamically remodelled to provide the structural platform for the development of membrane protrusions such as filopodia and lamellipodia which are implicated in the adhesion to the extracellular matrix and in the generation of the cell's locomotive force [18].

This process is regulated by several intermediates, including the ezrin/radixin/moesin (ERM) family of actin-binding proteins [19]. We recently showed that 17β-estradiol (E2) leads to dynamic rearrangements of the actin cytoskeleton and promotes cell migration via the activation of moesin in human endothelial cells [20], suggesting that the ERM protein-mediated actin remodeling represents a privileged target of sex steroids for the control of cell movement.

In this manuscript we investigate the differential effects of natural progesterone and of the synthetic progestins MPA, DRSP and NES, alone or in combination with E2, on moesin activation, actin remodeling, cell migration and invasion in T47-D breast cancer cells.

## Methods

### Cell cultures and treatments

T47-D and MCF-7 breast cancer cells were incubated in DMEM (GIBCO) containing 10% fetal calf serum (FCS) and 0.2 UI/mL insulin, L-glutamine, penicillin and streptomycin under a 5% CO_2 _atmosphere at 37°C. MDA-MB-468 breast cancer cells were incubated in L-15 medium (Leibovitz)(GIBCO) containing 10% fetal calf serum (FCS) and L-glutamine, penicillin and streptomycin. Before treatments, cells were kept 48 hours in DMEM containing steroid-deprived FBS. Before experiments investigating non-transcriptional effects, the cells were kept in DMEM containing no FBS for 8 hours. Whenever an inhibitor was used, the compound was added 30 minutes before starting the treatments. Progesterone, medroxyprogesterone acetate, 17β-estradiol, PTX, Y-27632, PD98059 and wortmannin were from Sigma-Aldrich (Saint-Louis, MO). Drospirenone was a kind gift of Dr. Heiner Fritzemeier (Bayer Schering Pharma, Berlin, Germany), Nestorone was provided by Dr. R. Sitruk-Ware and ORG 31710 was a kind gift of Dr. Lenus Kloosterboer, from Organon Akzo Nobel (Oss, The Netherlands).

### Cell immunofluorescence

T47-D breast cancer cells were grown on coverslips and exposed to treatments. The cells were fixed with 4% paraformaldehyde for 30 min and permeabilized with 0.1% Triton for 5 min. Blocking was performed with PBS containing 1% bovine serum albumin for 30 min. Then cells were incubated with Texas Red-phalloidin (Sigma) for 10 min. After washing the nuclei were counterstained with 4'-6-diamidino-2-phenylindole (DAPI) (Sigma) and mounted with Vectashield mounting medium (Vector Laboratories, Burlingame, CA). Immunofluorescence was visualized using an Olympus BX41 microscope and recorded with a high-resolution DP70 Olympus digital camera. Cell membrane thickness and the gray level of extracellular area, cell membrane as well as cytoplasm were quantitated using Leica QWin image analysis and image processing software (Leica Microsystems, Wetzlar, Germany).

### Immunoblottings

Cells were harvested in lysis buffer including100 mM Tris-HCl (pH 6.8), 4% SDS, 20% glycerol, 1 mM Na_3_VO_4_, 1 mM NaF, and 1 mM PMSF. Cell lysates were separated by SDS-PAGE. The antibodies used were: moesin (clone 38, Transduction Laboratories, Lexington, KY), Thr^558^-P-moesin (sc-12895, Santa Cruz Biotechnology, Santa Cruz, CA). Primary and secondary Abs were incubated with the membranes with standard technique [21]. Immunodetection was accomplished using enhanced chemiluminescence.

### Transfection experiments

Plasmids for CMV human progesterone receptor A (hPR-A, # 95) and B (hPR-A, # 90) were provided by Dean P. Edwards (Baylor college of medicine, USA). Both plasmids (15 μg) were transfected into MDA-MB-468 breast cancer cells using the Lipofectamine (Invitrogen) according to the manufacturer's instructions. Cells (60–70% confluent) were treated 48 h after transfection and prepared according to the experiments to be performed.

Validated antisense phosphorotioate oligonucleotides (S-modified) (PONs) complementary to the 1–15 position of the human moesin gene coding region were obtained from Dharmacon. The sequence was 5'-TACGGGTTTTGCTAG-3' for moesin antisense PON. The complementary sense PON was used as control (5'-CTAGCAAAACCCGTA-3'). Transfections were performed on subconfluent T47-D cells. PONs were resuspended in serum-free medium with Lipofectamine (Invitrogen) and added to the culture medium every 12 h at the final concentration of 4 μM. Every 24 h, cells were washed and fresh medium supplemented with 4 μM PONs was added. Moesin silencing was assessed through protein analysis up to 48 h after transfection.

### Cell migration assays

Cell migration was assayed with razor scrape assays as previously described [20]. Briefly, a razor blade was pressed through the confluent T47-D breast cancer cell monolayer into the plastic plate to mark the starting line. T47-D cells were swept away on one side of that line. Cells were washed, and 2.0 mL of DMEM containing steroid-deprived FBS and gelatin (1 mg/mL) were added. Cytosine β-D-arabinofuranoside hydrochloride (Sigma) (10 μM), a selective inhibitor of DNA strand separation which does not inhibit RNA synthesis was used 1 h before the test substance was added. Migration was monitored for 48 hours. Every 12 h fresh medium and treatment were replaced. Cells were digitally imaged and migration distance was measured by using phase-contrast microscopy.

### Cell invasion assays

Cell invasion were assayed following the standard method by using the BD BioCoatTM Growth Factor Reduced (GFR) Matrigel™ Invasion Chamber (BD Bioscience, USA). In brief, after rehydrating the GFR Matrigel inserts, the test substance was added to the wells. An equal number of Control Inserts (no GFR Matrigel coating) were prepared as control. 0.5 mL of T47-D cell suspension (2.5 × 10^4 ^cells/mL) was added to the inside of the inserts. The chambers were incubated for 24 h at 37°C, 5% CO_2 _atmosphere. After incubation, the non-invading cells were removed from the upper surface of the membrane using cotton tipped swabs. Then the cells on the lower surface of the membrane were stained with Diff-Quick stain. The invading cells were observed and photographed under the microscope at 100× magnification. Cells were counted in the central field of triplicate membranes. The invasion index was calculated as the % invasion test cell/% invasion control cell.

### Statistical analysis

All values are expressed as mean ± SD. Statistical differences between mean values were determined by ANOVA, followed by the Fisher's protected least significance difference (PLSD).

## Results

### Effects of P, MPA, DRSP and NES on the actin cytoskeleton

**Figure 1:**
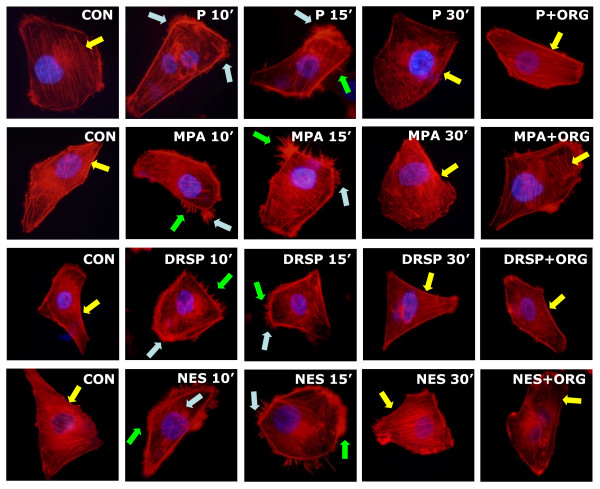
**Progestins induce a rapid rearrangement of the actin cytoskeleton in T47-D cells. **T47-D cells were treated with P, MPA, DRSP (all 100 nM) or NES (1 nM) for 10, 15 or 30 minutes, in the presence or absence of the pure PR antagonist ORG 31710 (1 μM). Immunofluorescent staining of actin (in red) reveals the spatial modifications of actin fibers through the time-course and the formation of specialized cell membrane structures (yellow arrows indicate longitudinal actin fibers, green arrows show pseudopodia, light blue arrows indicate ruffles). Nuclei are counterstained in blue.

Our first objective was to identify the effects of P, MPA, DRSP and NES on the spatial organization of actin fibers. We thus exposed steroid- and serum-deprived T47-D (ER^+^/PR^+^) breast cancer cells to these compounds and stained the actin cytoskeleton with phalloidin linked to a fluorescent dye (Texas Red). Based on the evidence that P, MPA and DRSP have comparable binding affinities for progesterone receptor (PR), while NES is about a 100-fold more effective than P in binding to PR [11], we used a 100-fold lower concentration of NES than other three progestins. At baseline, actin fibers were arranged longitudinally through the major axis of T47-D cells, which displayed regular cell borders (Fig. 1). In the presence of any of the four progestins (P, MPA, DRSP all 100 nM; NES, 1 nM), the vast majority of breast cancer cells displayed visible changes of actin fibers organization (within 10 to 15 min), with a rapid actin concentration at the cell membrane (Fig. 1). This was associated with a significant increase of the thickness of the cell membrane and of its fluorescence intensity, quantified by analyzing the pixel intensity including the cell membrane as well as the adjacent intra- and extra-cellular space (Table 1). Within the same time frame, cell membrane structures involved in cell adhesion and movement, including ruffles, focal adhesion complexes and pseudopodia were formed (Fig. 1). This process was transient, with actin fibers going back to the basal arrangement within 30 minutes, and was blocked by the PR antagonist ORG 31710 (1 μM), indicating the involvement of PR (Fig. 1).

**Table 1:** Membrane modifications after hormone treatments in different breast cancer cell lines

	Treatment (15 min)	Mean membrane thickness (pixel ± SD)(× 1000)	Mean membrane intensity (mean gray level ± SD)	Mean cytosol intensity (mean gray level ± SD)	% cells with actin modifications
T47-D	CON	32.4 ± 5.8	61.5 ± 8.3	59.8 ± 5.6	5.2 ± 0.8 %
	P	62.3 ± 9.2*	102.2 ± 10.4*	62.3 ± 7.4	64.5 ± 5.4 % *
	P+E2	78.2 ± 10.3*	124.5 ± 13.2*	58.5 ± 6.1	72.2 ± 5.7 % *
	MPA	76.3 ± 14.6*	118.6 ± 12.7*	67.8 ± 8.2	69.6 ± 6.1 % *
	MPA+E2	92.5 ± 12.3*	131.8 ± 12.4*	57.4 ± 6.6	81.8 ± 4.9 % *
	DRSP	54.6 ± 6.7 *	87.2 ± 8.3 *	61.6 ± 8.2	60.4 ± 4.4 % *
	DRSP+E2	60.3 ± 5.2 *	98.5 ± 11.6*	57.8 ± 6.7	68.1 ± 5.2 % *
	NES	71.5 ± 8.6*	110.2 ± 14.4 *	61.4 ± 8.5	69.6 ± 6.5 % *
	NES+E2	80.6 ± 10.5 *	127.3 ± 7.2 *	59.1 ± 5.4	73.1 ± 5.6 % *
MCF-7	CON	46.6 ± 4.7	77.8 ± 5.8	74.3 ± 6.2	6.7 ± 1.3 %
	P	99.2 ± 7.4 *	132.4 ± 8.6 *	68.5 ± 4.7	70.6 ± 7.4 % *
	P+E2	115.8 ± 8.3 *	140.3 ± 10.2 *	66.3 ± 5.2	68.3 ± 6.2 % *
	MPA	108.4 ± 11.3 *	142.7 ± 12.5 *	60.4 ± 5.6	78.2 ± 7.8 % *
	MPA+E2	123.6 ± 10.8 *	150.2 ± 14.6 *	62.5 ± 4.9	80.5 ± 7.2 % *
MDA-MB-468	CON	31.2 ± 4.4	66.8 ± 7.2	62.6 ± 5.5	5.4 ± 0.7 %
	P	34.3 ± 4.1	70.5 ± 6.4	64.2 ± 5.8	4.8 ± 0.6 %
	P+E2	32.5 ± 3.9	68.1 ± 5.8	61.4 ± 4.3	5.1 ± 0.4 %
	MPA	36.2 ± 5.2	67.2 ± 5.5	70.5 ± 5.3	4.4 ± 0.8 %
	MPA+E2	32.2 ± 4.6	63.6 ± 6.2	66.3 ± 5.6	5.2 ± 0.7 %
MDA-MB-468 (transfected with PR)	CON	34.6 ± 4.3	65.4 ± 6.8	70.4 ± 5.2	5.2 ± 0.9 %
	P	66.7 ± 6.8 *	97.4 ± 8.3 *	66.3 ± 4.8	58.6 ± 6.7 % *
	P+E2	72.5 ± 7.1 *	106.3 ± 10.6 *	72.5 ± 6.4	60.3 ± 6.9 % *
	MPA	70.4 ± 7.4 *	102.8 ± 11.8 *	68.2 ± 5.5	60.2 ± 6.6 % *
	MPA+E2	76.2 ± 8.2 *	118.5 ± 13.2 *	71.8 ± 6.8	65.8 ± 7.1 % *

The table displays the mean thickness of the cell membrane, the mean actin intensity of the membrane and of the cytoplasm, as well as the % cells with spatial actin modifications in several breast cancer cell lines treated with different regimens of hormones (P, MPA, DRSP, all 100 nM; NES, 1 nM; E2, 10 nM) for 15 min. Analytic results were obtained by using Leica QWin image analysis and processing software.

### Effects of P, MPA, DRSP and NES on actin remodeling in the presence of E2

**Figure 2:**
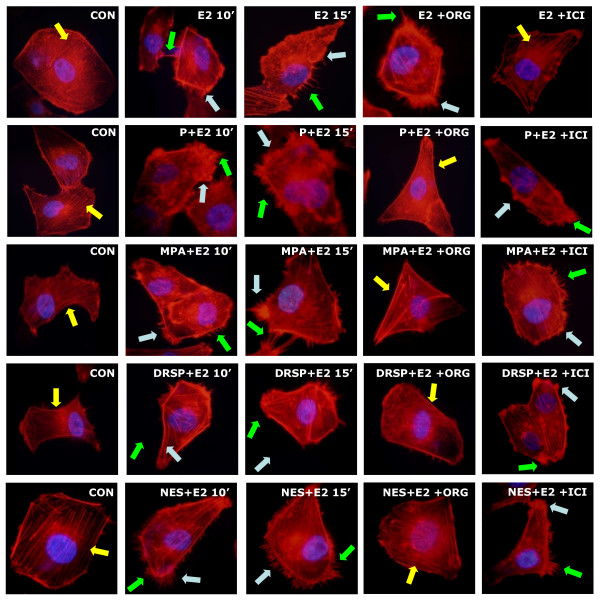
**Effects of progestins on actin remodeling in the presence of E2. **T47-D cells were treated with E2 (10 nM) or E2 (10 nM) plus P, MPA, DRSP (all 100 nM) or NES (1 nM) for 10 or 15 minutes, in the presence or absence of the pure PR antagonist ORG 31710 (ORG – 1 μM) and the pure ER antagonist ICI 182,780 (100 nM). Yellow arrows indicate longitudinal actin fibers, green arrows show pseudopodia, light blue arrows indicate ruffles. Nuclei are counterstained in blue.

E2 (10 nM) rapidly induced actin rearrangement in T47-D breast cancer cells, consistent with its action on endothelial cells [20]. Each progestin, when added to E2, did not significantly change the effect of E2 itself, although the cells often display a somewhat more evident rearrangement of actin fibers (as shown by the quantification of the mean thickness and intensity of cell membrane, Table 1) and cell membrane structures formation as compared to treatment with the progestin alone (Fig. 2). The effect of the addition of the progestins to E2 was reduced by the PR antagonist ORG 31710 (1 μM) but not by the ER antagonist ICI 182,780 (100 nM) (Fig. 2).

**Figure 3:**
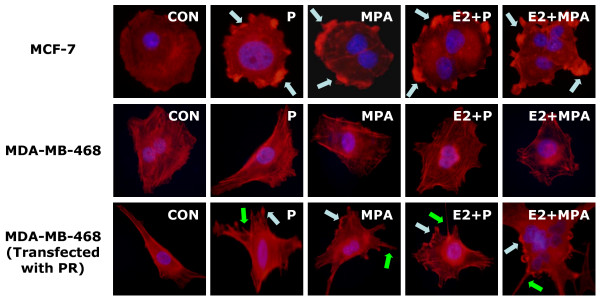
**MCF-7 cells and MDA-MB-468 cells with or without PR transfection were treated with P and MPA (both 100 nM), in the presence or absence of E2 (10 nM).** Yellow arrows indicate longitudinal actin fibers, green arrows show pseudopodia, light blue arrows indicate ruffles. Nuclei are counterstained in blue.

Likewise, in ER/PR positive MCF-7 breast cancer cells, P and MPA (both 100 nM) also provoked the rapid actin reorganization in the absence or presence of E2 (10 nM), which were indicated by the translocation of actin towards cell membrane and by the obvious formation of lamellipodia (Fig. 3, Table 1). However, in MDA-MB-468 ER^-^/PR^- ^breast cancer cells, the same compounds failed to induce actin cytoskeleton remodeling (Fig. 3, Table 1). When MDA-MB-468 cells were transfected with plasmids encoding PRA and PRB, they became able to respond to progestin exposure with actin rearrangement (Fig. 3, Table 1), confirming the central role of PR.

### Comparative effects of P, MPA, DRSP and NES on the actin-regulatory protein, moesin

**Figure 4:**
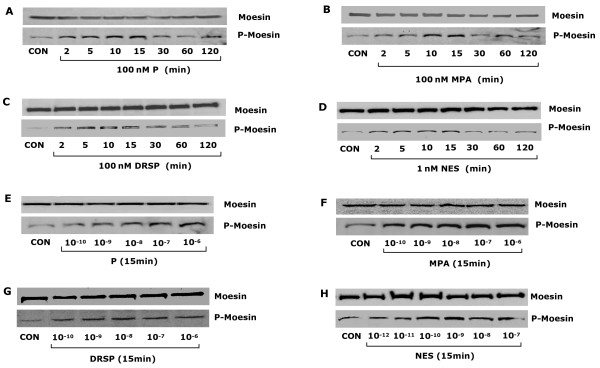
**Progestins activate moesin.** (A-H) show total cell amount of wild-type moesin or Thr^558^-phosphorylated moesin (P-Moesin) in cells treated with the indicated progestins at different time points or concentrations.

Treatment of T47-D cells with P, MPA, DRSP (all 100 nM) or NES (1 nM) resulted in rapid increases of Thr^558^-phosphorylation of moesin (which corresponds to activation) [22], with a first visible effect from 2 minutes, a phosphorylation peak at 15 minutes, and a progressive decline to basal levels between 30 minutes and 2 hours, which is time-consistent with the kinetics of actin rearrangement (Fig. 4A–D). The amount of phosphorylated moesin was related to the concentration of the compounds (Fig. 4E–H). In parallel, the cell content of wild-type moesin did not change during this time frame (Fig. 4A–H).

**Figure 5:**
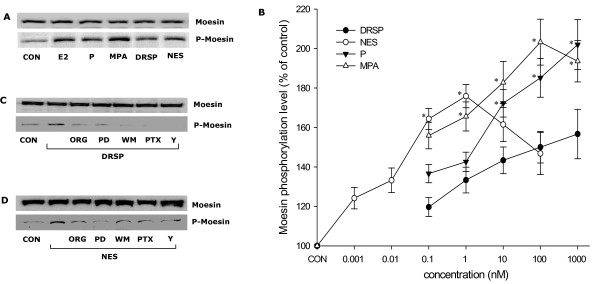
**Comparative effects of different progestin on moesin activation.** (A) T47-D breast cancer cells were treated with E2 (10 nM) or progestins (P, MPA, DRSP all 100 nM; NES, 1 nM) for 15 min and total cell amount of Moesin or P-Moesin are shown. (B) Shows the concentration/effect curve of each progestin on moesin phosphorylation over a range of concentrations. * = P < 0.05 vs. DRSP at the same concentration. (C-D) Cells were exposed to DRSP (100 nM) or NES (1 nM) for 15 min, in the presence or absence of the pure PR antagonist ORG 31710 (ORG – 1 μM), of the MEK inhibitor PD98059 (PD – 5 μM) or of the PI3K inhibitor wortmannin (WM – 30 nM), of the G protein inhibitor, PTX (100 ng/mL) or of the ROCK-2 inhibitor Y-27632 (10 μM). Total cell amounts of Moesin or P-Moesin are shown.

When bioequivalent amounts of P, MPA, DRSP (all 100 nM) or NES (1 nM) were administered to T47-D cells, visible differences in moesin Thr^558^-phosphorylation were seen, with MPA inducing the strongest activation and DRSP the weakest (Fig. 5A). In order to more precisely compare the potency of each progestin on moesin activation, we performed dose-response curves of moesin phosphorylation with the four compounds by quantitatively analysing Thr^558^-phosphorylated moesin bands intensities with a chemiluminescence digital acquisition system. As shown in Fig 5B, MPA was consistently more potent in inducing moesin activation as compared to P and DRSP. DRSP was significantly less effective than P or MPA (Fig. 5B). As expected, the dose-response curve of NES was shifted to the left by two orders of magnitude compared with P, MPA or DRSP (Fig 5B). However, if bio-equivalent concentrations are compared (1 nM NES vs. 100 nM P or 0.1 nM NES vs. 10 nM P), NES shows comparable effects on moesin as P, being less active than MPA (Fig 5B).

### Signaling mechanisms of DRSP and NES to moesin

We recently identified that natural P triggers moesin phosphorylation through a PRA/Gα_13_/RhoA/Rho-associated kinase (ROCK) pathway, while MPA recruits a PR/Src/phosphatidylinositol-3-kinase (PI3K)/RhoA/ROCK cascade (X.D. Fu et al, submitted). Moesin phosphorylation induced by DRSP (100 nM) or NES (1 nM) was prevented by the PR antagonist ORG 31710 (1 μM), by the G protein inhibitor pertussis toxin (PTX – 100 ng/mL) and by Y-27632 (10 μM), a specific inhibitor of ROCK-2, which is a known activator of moesin (Fig. 5C–D). However, distinct from the signaling pathways exploited by P and MPA, the MAPK inhibitor PD98059 (5 μM) and the PI3K inhibitor wortmannin (30 nM) also interfered with moesin activation by DRSP (100 nM) and NES (1 nM) (Fig. 5C–D), suggesting that MAPK and PI3K play a role in moesin activation by these progestins, and supporting the concept that each progestin might recruit partly distinct PR-dependent signaling cascades.

### Comparative effects of P, MPA, DRSP and NES on moesin activation in the presence of E2

**Figure 6:**
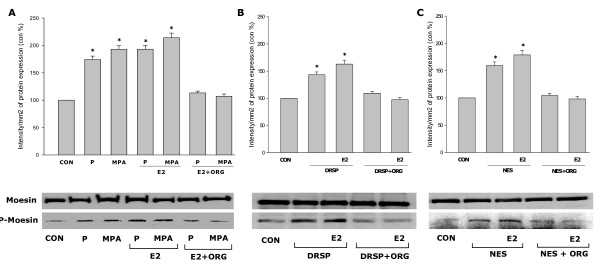
**Effects of progestins on moesin activation in the presence of E2.** (A-C) T47-D breast cancer cells were exposed to each progestin alone (P, MPA, DRSP all 100 nM; NES, 1 nM) or together with E2 (10 nM) for 15 min in the presence or absence of ORG 31710 (ORG – 1 μM). Total cell amounts of Moesin or P-Moesin are shown.

Estrogen signals to moesin through rapid, extra-nuclear signaling [20]. Moesin phosphorylation was slightly increased by the addition of E2 (10 nM) to each progestin compared to the progestins alone, although this was not statistically significant (Fig. 6A–C). Interestingly, the PR antagonist ORG 31710 (1 μM) inhibited the combined effect of E2 associated with each progestin on moesin (Fig. 6A–C).

### Comparative effects of P, MPA, DRSP and NES on breast cancer cell migration and invasion

**Figure 7:**
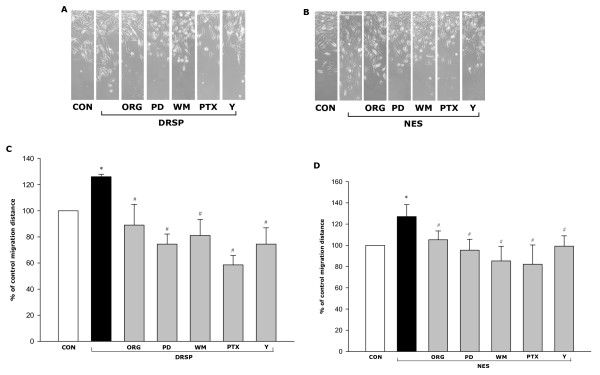
**DRSP and NES promote T47-D breast cancer cell horizontal migration.** (A-B) Cells were treated with DRSP (100 nM) or NES (1 nM) for 48 h, in the presence or absence of ORG 31710 (ORG – 1 μM), of PD98059 (PD – 5 μM), of wortmannin (WM – 30 nM), of PTX (100 ng/mL) or of Y-27632 (Y – 10 μM). T47-D cells were scraped out of the cell culture dish and the extent of migration of the remaining cells was assayed in non-proliferating cells in the presence of Ara-C. Representative images of cell migration are shown. (C-D) Cell migration distances were measured and values are presented as % of control. * = P < 0.05 vs. control; # = P < 0.05 vs. DRSP or NES; The experiments were performed in triplicates and data representing the migration distance of cells from the starting line are expressed as mean ± SD.

Our recent observation indicates that P and MPA promote T47-D cell horizontal migration through a complex cascade requiring PR, G proteins, MAPK, PI3K and the Rho-associated kinase, ROCK-2 (X.D. Fu et al, submitted). DRSP (100 nM) and NES (1 nM) increased T47-D cell migration, alike (Fig. 7A–D). DRSP- or NES-promoted cell migration were reduced by ORG 31710 (1 μM), by PTX (100 ng/mL), by the MAPK inhibitor PD98059 (5 μM), by the PI3K inhibitor wortmannin (30 nM) and by the ROCK inhibitor Y-27632 (10 μM) (Fig. 7A–D).

**Figure 8:**
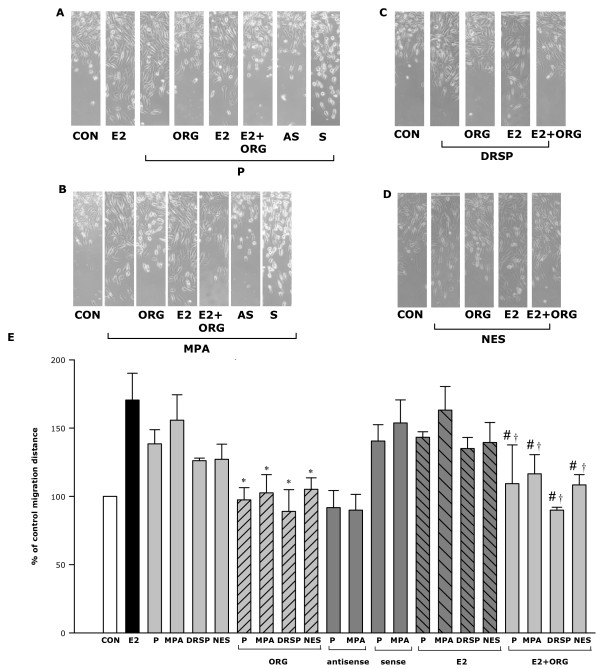
**Effects of progestins in combination with E2 on T47-D breast cancer cell migration.** (A-D) Cells were treated with the progestins (P, MPA, DRSP all 100 nM; NES, 1 nM) alone or in combination with E2 (10 nM) for 48 h, in the presence or absence of ORG 31710 (ORG – 1 μM). Other cells were transfected with moesin antisense phosphorotioate oligonucleotides (PON) (AS, antisense – 2 μM) or sense PON (S, sense – 2 μM). T47-D cells were scraped out of the cell culture dish and the extent of migration of the remaining cells was assayed in non-proliferating cells in the presence of Ara-C. Representative images of cell migration are shown. (E) Cell migration distances were measured and values are presented as % of control. * = P < 0.05 vs. the corresponding progestin alone; # = P < 0.05 vs. E2 + the corresponding progestin without ORG; † = P < 0.05 vs. E2.

We next compared the potency of these progestins on breast cancer cell migration. Among the tested compounds, MPA (100 nM) was the most potent, increasing cell migration by 54% compared to control (Fig. 8A–E). P (100 nM), DRSP (100 nM) and NES (1 nM) increased T47-D horizontal migration vs. control by 37%, 32% and 26% (Fig. 8A–E), respectively, which agrees with the order they rank on moesin activation.

In order to check for the requirement of moesin for progestins-promoted cell migration we silenced moesin with antisense oligonucleotides (PONs) (Fig 8A, 8B, 8E). Transfection with moesin antisense PONs greatly reduced the action of both progesterone and MPA on cell migration, while sense PONs had no impact (Fig. 8A, 8B, 8E).

E2 promoted T47-D cell migration as well. However, no significant additive effects were found during co-treatment with any of the progestins. Interestingly, the PR antagonist ORG31710 (1 μM) significantly reduced cell migration associated with the four tested progestins both in the absence and in the presence of E2 (Fig. 8A–E).

**Figure 9:**
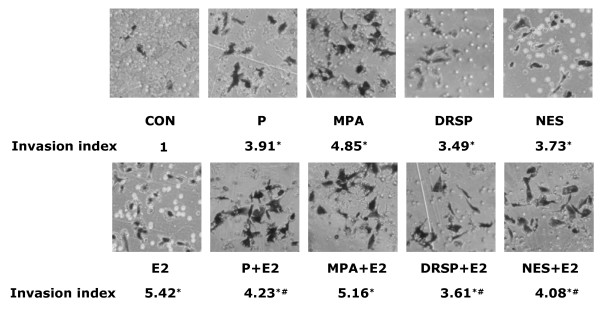
**Progestins alone or in combination with E2 promote T47-D breast cancer cell invasion. **Cells were treated with the progestins alone (P, MPA, DRSP all 100 nM; NES, 1 nM) or in combination with E2 (10 nM) for 24 h and cell invasion was assayed with matrigel invasion assays. Invading cells were counted in three different central fields of triplicate membranes. Invasion indexes and representative images are shown. * = P < 0.01 vs. control; # = P < 0.05 vs. E2.

Finally, we investigated the actions of the four progestins on breast cancer cell invasion of a three-dimensional matrix (matrigel). P, MPA, DRSP (all 100 nM) and NES (1 nM) all enhanced cell invasive behavior (Fig. 9). Consistently with the previous findings, the invasion indexes indicate that MPA is more effective compared to the other compounds (invasion index 4.68). Treatment with P, NES and DRSP resulted in invasion indexes of 3.91, 3.73 and 3.49, respectively. E2 (10 nM) was more potent than the progestins in driving breast cancer cell invasion of the matrix. When T47-D cells were exposed to E2 plus P, DRSP or NES the invasion indexes turned out to be significantly reduced vs. treatment with E2 alone. The addition of MPA to E2 also resulted in a reduction of the invasion index below that of E2 alone, but this did not reach statistical significance (Fig. 9).

## Discussion

The role of progestins on breast cancer development or progression is controversial [23]. A variety of progestins are currently used in postmenopausal HRT and circumstantial evidence from recent clinical trials suggests that each compound may differently contribute to the risk of developing breast cancer [5]. However, limited information is available on the impact of progestins on breast cancer progression.

The present work shows that four different progestins, including natural progesterone, the synthetic progestin MPA, and two newer progestins, DRSP and NES, all enhance PR+ breast cancer cell migration and invasion *in vitro*. These effects are coupled to the activation of the actin-binding protein moesin, which drives actin fibers to the cell membrane, increasing the formation of specialized membrane structures which interact with the extracellular matrix and with nearby cells, thus allowing the cells to achieve locomotion.

The control of intracellular actin organization by progestins represents an original mechanism through which these hormonal compounds may alter the ability of breast cancer cells to move. This adds to the previously reported effects of progesterone on breast cancer cell invasion through tyrosine phosphorylation of focal adhesion kinase [24], through increased tissue factor gene expression or glucose uptake [25,26], or through the activation of matrix metalloproteinases and urokinase-type plasminogen activator [27]. These findings, along with ours, identify potential targets for the development of drugs against breast cancer progression linked to steroids, particularly endogenous or exogenous progestogens.

Progestins exert their biological functions principally by binding to PR [28]. In the present study, the pure PR antagonist ORG 31710 blocks moesin activation, cell migration and invasion induced by all four compounds, supporting the central role of PR in these processes. In agreement, PR negative cell lines show actin remodeling in response to progestins only after transfection with PR.

However, the spectrum of promiscuous binding to other steroid receptors varies significantly among progestins, leading to variable cellular effects. In addition to this, distinct signaling pathways can be recruited by PRs in the presence of different ligands. We recently showed that P and MPA induce the recruitment of partially distinct signaling cascades in endothelial cells acting on PRs [29] and we recently identified similar differences in breast cancer cells (Fu XD, et al. submitted). Our present findings strengthen this concept, suggesting that depending on the ligand, PRs may be driven to recruit different signal transduction pathways to accomplish multiple functions in human cells.

The progestational potency of each compound is usually compared by identifying the dose associated with full endometrial transformation and ovulation inhibition in animals [2]. However, the real progestogenic activity depends on a variety of factors, including the route and timing of administration and the specific endpoint tested, such that it is difficult to definitely establish the most appropriate concentration to compare two progestins. In the present study, we performed concentration-dependence curves to better compare the effects of these progestins over a wider range of concentrations on some of the investigated targets. Our results indicate that the order of potency for the studied actions in T47-D breast cancer cells is MPA > P > DRSP. Given the fact that NES is 100 times more effective than P in transforming the endometrium and in binding PRs [11], we selected a 100-time lower concentration for comparative analyses. When comparing these concentrations, NES exerts effects which are comparable to those of P on moesin activation. Overall, these findings suggest that markers of biological/functional effect, such as moesin activation or cell migration and invasion might be of relevance to better characterize the comparative actions of progestins in pre-clinical settings.

Interestingly, our findings show that the combination of P, DRSP or NES with E2 turns into a significant decrease of cell invasion vs. E2 alone. This is not found for the combination of MPA with E2. However, this interference with estrogen-dependent cell invasion displayed by P, NES and DRSP is not related to the regulation of moesin or of the actin cytoskeleton. These findings suggest that, notwithstanding that both estrogen and progestins promote T47-D breast cancer cell migration and invasion, some progestins partially offset the effect of E2 on ER+/PR+ breast cancer cell invasion, but also that this does not extend to all PR ligands.

This apparent discrepancy could be ascribed to the different molecular actions of sex steroids involved in these processes [30]. Indeed, moesin activation and actin remodeling are recruited though rapid, extra-nuclear signaling pathways of ER and PR, while the regulatory effects on cell migration and invasion likely derive from complex integrations of nongenomic and genomic actions. Indeed, the recruitment of ER and PR each turns into the regulation of a complex subset of target genes, whose function on cell movement or invasion is not yet investigated. Moreover, recent findings indicate that PR may act as an ER antagonist in certain circumstances, altering the ability of ER to interact with estrogen response elements and to trigger gene expression [31].

Blockade of PR with ORG 31710 inhibits moesin activation and cell migration induced by the combination of E2 with each progestin. A similar observation has already been reported in breast cancer cells, where the up-regulation of breast cancer resistance protein expression induced by the combination of E2 plus progesterone is abolished by the progesterone receptor antagonist RU486 [32]. One possible explanation of this observation could be that PR and ER need to be cross-coupled to induce this action, and that the presence of a PR agonist might facilitate this phenomenon. On the opposite, the presence of the PR antagonist may interfere with the ability of PR to interact with ER, thus antagonizing the function of both ER and PR. This would be consistent with the established ability of the ER antagonist ICI 182,780 to block PR signaling in breast cancer cells [33] but additional studies will be necessary to provide a definitive answer.

## Conclusion

Taken together, our findings show that P, MPA, DRSP and NES alone or in combination with E2 increase breast cancer cell migration and invasion through the functional modulation of the actin-binding protein moesin and the induction of dynamic rearrangements of the actin cytoskeleton. This suggests that progestins may have an impact on the progression of PR+ breast cancer by altering the ability of cancer cells to interact with the extracellular environment and to eventually move or invade the surrounding environment. The potency of the progestins on these targets is however different, with maximal effects induced by MPA, followed by P, NES and DRSP. These differences in biological efficacy are possibly related to partially discrepant recruitment of extra-nuclear signaling pathways by PR in the presence of each progestin. All together, these findings provide evidence that PR activation might play a role in the progression of ER+/PR+ breast cancers.

## Abbreviations

Progesterone: P; Medroxyprogesterone acetate: MPA; Drospirenone: DRSP; Nestorone: NES; Progesterone receptor: PR; 17β-estradiol: E2; Pertussis toxin: PTX; Mitogen activated protein kinase: MAPK; phosphatidylinositol-3-kinase: PI3K; Rho-associated kinase: ROCK.

## Competing interests

The authors declare that they have no competing interests

## Authors' contributions

XDF, designed and carried out the experiments, analyzed the data, drafted and revised the manuscript; MsG, LG, MF, AMS, CB, SG, carried out the experiments; RS-W, helped to revise the manuscript; ARG, reviewed and revised the manuscript; TS, designed the experiments, analyzed the data, drafted and revised the manuscript. All authors read and approved the final manuscript.

## Pre-publication history

The pre-publication history for this paper can be accessed here:


